# The prognostic marker KRT81 is involved in suppressing CD8 + T cells and predicts immunotherapy response for triple-negative breast cancer

**DOI:** 10.1080/15384047.2024.2355705

**Published:** 2024-05-22

**Authors:** Zhideng Yan, Zhihui Zhong, Chuanke Shi, Muyin Feng, Xiaoqiang Feng, Tong Liu

**Affiliations:** a Department of General Surgery, Zhongshan Hospital of Traditional Chinese Medicine Affiliated to Guangzhou University of Traditional Chinese Medicine, Zhongshan, Guangdong, China; b Center of Stem Cell and Regenerative Medicine, Gaozhou People’s Hospital, Gaozhou, Guangdong, China; c Department of Pathology, Zhongshan Hospital of Traditional Chinese Medicine Affiliated to Guangzhou University of Traditional Chinese Medicine, Zhongshan, Guangdong, China

**Keywords:** Triple-negative breast cancer, keratin 81, prognostic gene, immunotherapy response, immune microenvironment, CD8 + T cells

## Abstract

Triple-negative breast Cancer (TNBC) is an aggressive subtype lacking estrogen, progesterone, and HER2 receptors. Known for limited targeted therapies, it poses challenges and requires personalized treatment strategies. Differential analysis revealed a significant decrease in keratin 81 (KRT81) expression in non-TNBC samples and an increase in TNBC samples, lower KRT81 expression correlated with better TNBC patient outcomes. It emerged as an independent predictive factor for TNBC, with associations found between its expression and clinically relevant features. We further developed a nomogram for survival probability assessment based on Cox regression results, demonstrating its accuracy through calibration curves. Gene annotation analysis indicated that KRT81 is involved in immune-related pathways and tumor cell adhesion. KRT81 is associated with immune cell infiltration of Follicular helper T cells (Tfh) and CD8 + T cells, suggesting its potential impact on the immunological microenvironment. The study delved into KRT81‘s predictive value for immunotherapy responses, high expression of KRT81 was associated with greater potential for immune evasion. Single-cell RNA sequencing analysis pinpointed KRT81 expression within a specific malignant subtype which was a risk factor for TNBC. Furthermore, KRT81 promoted TNBC cell proliferation, migration, invasion, and adhesion was confirmed by gene knockout or overexpression assay. Co-culture experiments further indicated KRT81‘s potential role in inhibiting CD8 + T cells, and correlation analysis implied KRT81 was highly correlated with immune checkpoint CD276, providing insights into its involvement in the immune microenvironment via CD276. In conclusion, this comprehensive study positions KRT81 as a promising prognostic marker for predicting tumor progression and immunotherapy responses in TNBC.

## GRAPHICAL ABSTRACT

**Figure:**
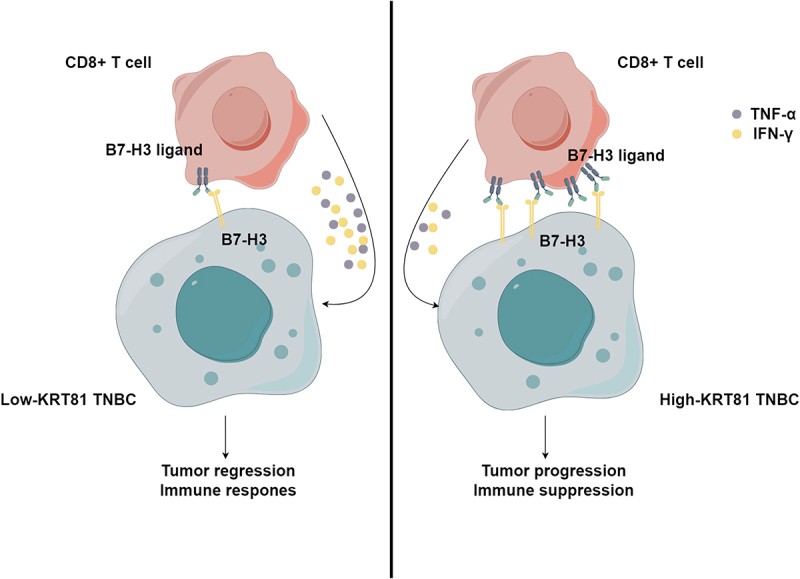

## Introduction

Breast cancer, a heterogeneous disease with diverse molecular subtypes, poses a significant health challenge globally.^1^ Among these subtypes, triple-negative breast cancer (TNBC) accounts for 10 to 15% of all breast cancer cases and stands out as a distinct and clinically challenging entity.^2^ TNBC is characterized by the absence of estrogen receptor (ER), progesterone receptor (PR), and human epidermal growth factor receptor 2 (HER2) expression, setting it apart from other breast cancer subtypes.^3^ This unique molecular profile not only distinguishes TNBC but also underscores its clinical complexity. The absence of these key receptors limits the application of targeted therapies commonly used in other breast cancer subtypes and may be diagnosed at a more advanced stage. This makes TNBC particularly difficult to treat, and traditional chemotherapy is often the mainstay of treatment. Hormone therapies that rely on ER and PR expression, as well as HER2-targeted therapies, find little efficacy in TNBC cases. As a result, TNBC often necessitates a different therapeutic approach, relying heavily on cytotoxic chemotherapy.^4^ The aggressive nature of TNBC, marked by a higher likelihood of metastasis and a faster rate of growth, further complicates treatment strategies.^3^ We aim to comprehensively explore the landscape of TNBC, delving into its molecular characteristics, and developing therapeutic targets that offer improved outcomes for individuals diagnosed with this aggressive breast cancer subtype.

The KRT family refers to a group of fibrous structural proteins that form the basis of certain tissues in the human body, particularly in the skin, hair, and nails that contribute to the structural organization of epithelial cells, forming cytoskeletal networks that provide mechanical support and resistance to cellular stress.^5,6^ In addition to their cytoskeletal functions, keratins are implicated in cellular processes such as cell motility, apoptosis, and signaling pathways.^7^ The diverse expression patterns of keratins across different tissues and cell types highlight their specificity in maintaining tissue integrity and function.^8^ While their primary function is related to cellular mechanical support and integrity, emerging research has illuminated their multifaceted involvement in cancer, particularly in the context of various epithelial malignancies, including breast cancer.^9,10^ Altered keratin expression patterns have been observed in various cancers, that are often associated with changes in cell morphology, invasiveness, and metastatic potential leading to tumor development and progression.^11^ The aberrant expression of keratin expression can serve as a diagnostic and prognostic marker in certain cancers, aiding in tumor classification and predicting clinical outcomes.^12^ Several members such as keratin 1, 7, 14, 19, and 20 have been reported to participate in the progression of TNBC via different pathways ^13–15^ However, little is known about the role of different members of the KRT family in triple-negative breast cancer.

The immune microenvironment refers to the complex interplay between the immune system and the surrounding tissue in a specific anatomical location, particularly within the background of diseases such as cancer.^16^ This microenvironment encompasses a dynamic network of immune cells, signaling molecules, and other components that collectively influence immune responses and regulate tissue homeostasis.^17^ In the context of cancer, the immune microenvironment plays a crucial role in determining the fate of tumor cells. It consists of various immune cell populations, such as T cells, B cells, natural killer cells, macrophages, and dendritic cells, as well as nonimmune components like fibroblasts and endothelial cells. The interactions within the immune microenvironment can either promote or inhibit tumor growth, invasion, and metastasis.^18^ The immune microenvironment in TNBC often exhibits complex interactions that can contribute to tumor progression. One notable aspect of the TNBC immune microenvironment is the presence of immunosuppressive elements, which can hinder the effective anti-tumor immune response.^19^ TNBC tumors may express immune checkpoint molecules, such as programmed death-ligand 1 (PD-L1). Interaction between PD-L1 on tumor cells and PD-1 on T cells can inhibit T cell function, leading to immune evasion by the tumor.^20^ Further, TNBC cells can actively secrete factors that contribute to immune evasion. This includes the production of immunosuppressive cytokines and the recruitment of immunosuppressive cells.^21–23^ Understanding the mechanisms of immunosuppression in the immune microenvironment of TNBC is critical for developing targeted therapies to overcome these barriers. Immunotherapeutic approaches, such as immune checkpoint inhibitors, aim to disrupt the immunosuppressive signals in the tumor microenvironment, unleashing the anti-tumor immune response.^24^ Ongoing research seeks to identify novel targets and combination strategies to enhance the effectiveness of immunotherapy in treating TNBC and improving patient outcomes. Nonetheless, it’s essential to note that not all TNBC patients will respond to immunotherapy, and the response rates can vary. It is urged to identify biomarkers for predicting immunotherapy response in TNBC.

## Results

### KRT81 is a potential prognostic marker for TNBC

**Figure 1.**
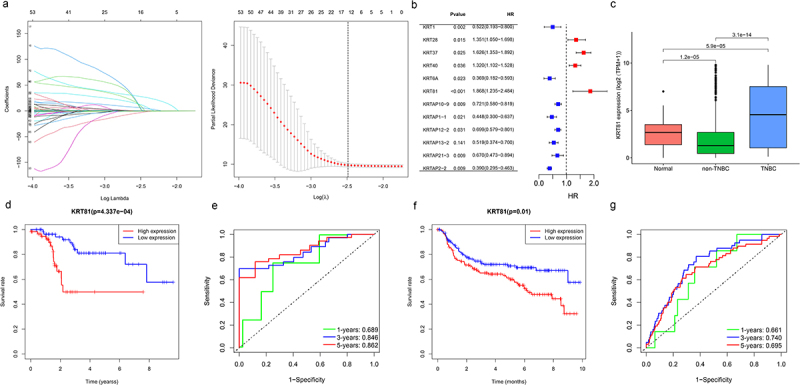
Identification of KRT81 as a valuable prognostic marker for TNBC. (a) Screening of optimal factors of the KRT family for prognosis prediction through LASSO Cox regression analysis. (b) Analyzing the HR value of the KRT-related prognostic genes by univariate Cox regression. (c) Comparison of the KRT81’s expression between the normal, non-TNBC, and TNBC samples. (d, e) the survival and ROC curves of KRT81 for predicting OS of TNBC in the TCGA cohort. (f, g) the survival and ROC curves of KRT81 for predicting OS of TNBC in the METABRIC cohort.

The analysis of the 150 collected KRT-related genes involved applying the LASSO Cox regression method to identify characteristic genes, resulting in the retention of 12 KRT-related genes (Figure 1(a)). Subsequently, the uniCox algorithm was employed to calculate the hazard ratio (HR) for these 12 genes, categorizing seven genes as protective factors (KRT1, KRT6A, KRTAP10–9, KRTAP1–1, KRTAP12–2, KRTAP13–2, KRTAP21–3, and KRTAP2–2) and four genes as risk factors (KRT28, KRT37, KRT40, and KRT81). Unexpectedly, KRT81 exhibited the highest HR among these genes (HR = 1.848, 95% CI: 1.235–2.484, *p* < .001) (Figure 1(b)). Further expanding our investigation, we assessed KRT81 expression levels across normal, non-TNBC, and TNBC samples in the TCGA cohort. The comparison revealed a noteworthy decrease in KRT81 expression within the non-TNBC group (logFC = −1.117, *p* = 1.2e-05), while a significant increase was observed in the TNBC samples, and was ranked sixth among the most significant upregulated genes (logFC = 2.354, *p* = 5.9e-05) (Figure 1(c)). We then proceeded to evaluate KRT81‘s prognostic relevance for TNBC patients using TCGA and METABRIC datasets through K-M plotter and ROC curves. In the TCGA cohort, TNBC patients with lower expression levels of KRT81 indicated a better prognosis (*p* = 4.337e-04, Figure 1(d)). The AUC values for 1-, 3-, and 5-year survival were 0.689, 0.846, and 0.862, respectively (Figure 1(e)). Similar significant results were obtained in the METABRIC database (Figure 1(f,g)). Moreover, we conducted a thorough examination to confirm the prognostic significance of KRT81 in predicting OS within the Luminal A, Luminal B, HER2 +, and TNBC subtypes via the K-M plotter online website. Elevated expression levels of KRT81 were found to be associated with a reduced survival time in Luminal A, HER2 +, and TNBC subtypes. Notably, the HR for KRT81 in TNBC was the most pronounced, reaching 2.79 (95% CI: 1.2–6.47, *p* = .0012) (Figure S1). These findings suggest that KRT81 could serve as a valuable prognostic marker for TNBC.

### KRT81 is an independent predicted factor for TNBC

**Figure 2.**
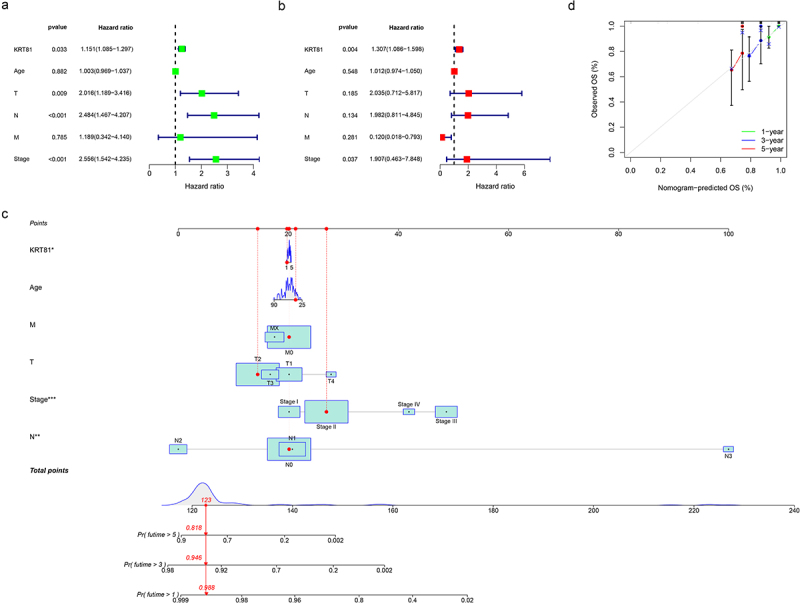
KRT81 prediction ability verification and construction of a nomogram. (a, b) Verification of the independent predictive ability of KRT81 via univariate and multivariate Cox regression analysis. (c, d) Construction and validation of a nomogram included clinical features and expression of KRT81 for predicting OS of TNBC patients.

We proceeded to conduct further investigations into the relationship between KRT81 expression and various clinically relevant features, such as age and TNM stage. As shown in Figure S2, in comparison to the T1 stage, KRT81 expression exhibited a decrease in the T3 stage (*p* = .015). When examining lymph node metastasis staging, the expression level of KRT81 was observed to be lower in the N3 stage compared to the N0 stage (*p* = .045). Additionally, KRT81 expression was associated with distant metastasis, showing upregulation in the M1 stage (*p* = .043). Further analysis revealed that KRT81 expression levels in stage II and stage III were significantly lower compared to stage I (*p* = .037 and *p* = .014, respectively). However, no statistical difference was observed between stage I and stage IV. To identify whether KRT81 could serve as an independent predictive factor from other clinical characteristics, we employed univariate and multivariate Cox regression methods. As shown in Figure 2(a), the hazard ratio value of KRT81 was 1.151 (95% CI: 1.085–1.297, *p* = .033). The outcomes of the multivariate Cox regression analysis also indicated that KRT81 stood as a significant risk factor for the OS of patients with TNBC (HR = 1.307, 95% CI: 1.086–1.598, *p* = .004) (Figure 2(b)). A quantitative assessment tool of the nomogram was formulated to evaluate the survival probabilities of TNBC based on the results of the Cox regression analysis (Figure 2(c)). Additionally, the calibration curves suggested that the tool had excellent capacity to predict the 1-, 3-, and 5-year OS rates (Figure 2(d)).

### Gene annotation and GSEA analysis

**Figure 3.**
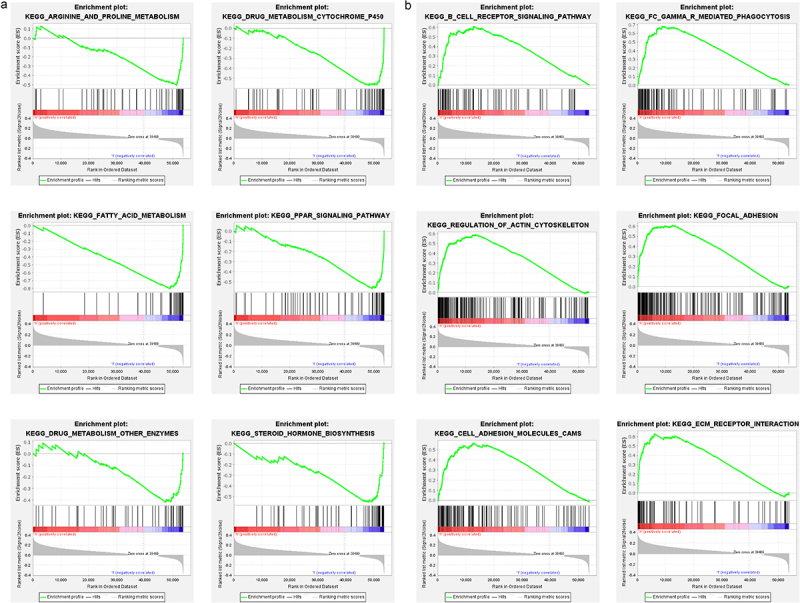
Functional annotation by GSEA algorithm. (a, b) the down- and up-regulated pathways involved in KRT81 were conducted by the GSEA method, respectively.

Subsequently, we explored the potential biological pathways and functions based on the differential expression genes (DEGs) among the high- and low-KRT81 groups via KEGG and GO analysis. A total of 145 DEGs were identified. The GO annotation distinctly revealed their predominant involvement in skin and muscle development. Furthermore, the pathways encompassing keratinization, muscle organ development, protein localization to the cell surface, keratin filament formation, and the structural constituents of the skin epidermis were significantly enriched (Figure S3). Concurrently, our KEGG analysis unearthed notable pathways such as folate biosynthesis, JAK-STAT signaling pathway, and focal adhesion. Notably, the breast cancer was also identified (Figure S3). Additionally, GSEA analysis showed that the KRT81-low group was enriched pathways of arginine and proline metabolism, drug metabolism cytochrome P450, fatty acid metabolism, PPAR signaling, drug metabolism of other enzymes, steroid hormone biosynthesis (Figure 3(a)). The KRT81-high group enriched pathways of immune-related pathways including B cell receptor signaling, Fc gamma Receptor-mediated phagocytosis, meanwhile, and the pathways of tumor cell progression such as regulation of actin cytoskeleton, focal adhesion, cell adhesion molecules cams, and ECM receptor interaction (Figure 3(b)). Collectively, our findings strongly suggest a potential correlation between elevated KRT81 expression levels and immunological activities, as well as cell adhesion and focal adhesion mechanisms in the context of TNBC.

### KRT81 correlates to immune cell infiltration in TNBC

**Figure 4.**
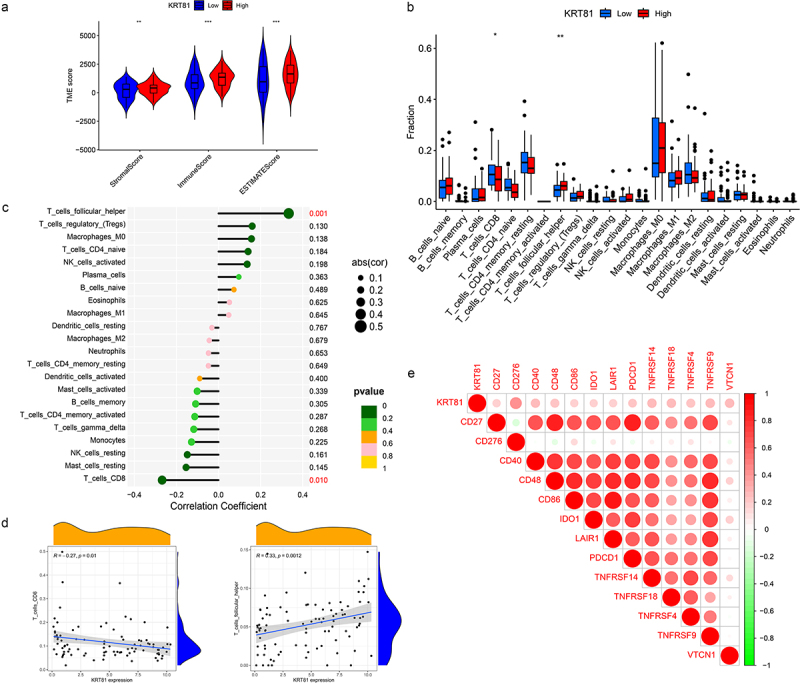
Correlation analysis of KRT81 and immune infiltration. (a) Differences in stromal, immune, and ESTIMATE scores between the high- and low-KRT81 groups were examined by the ESTIMATE method. (b) Differences in 22 types of immune cell infiltration were calculated via the CIBERSORT algorithm. (c, d) the correlation of KRT81 expression and immune cell infiltration. (e) The correlation analysis between KRT81 expression and immune checkpoints. The larger the circle, the higher the gene expression. The redder the color, the more significant the difference.

We further investigated the association of KRT81 and immune cell infiltration in TNBC. Initially, we adopted the ESTIMATE package to compute the stromal and immune scores. The results indicated that the high-KRT81 group exhibited elevated scores of stromal and immune (Figure 4(a)). Then, the CIBERSORT method was utilized to calculate the proportions of 22 different types of immune cells infiltrated in cancer. The results showed that follicular helper T (Tfh) cells were higher in the high-KRT81 group (*p* = .037), but there was a lower level of naïve CD8 + T cell infiltration (*p* = .011) (Figure 4(b)). Spearman analysis showed that the naïve CD8 T cell fraction decreased with KRT81 expression (cor = 0.27, *p* = .01), while the Tfh cell showed a reverse relationship (cor = 0.33, *p* = .0001) (Figure 4(c,d)). In addition, we performed an analysis to investigate the relationship between the expression of immune checkpoints and KRT81. The results revealed a positive correlation between KRT81 and several checkpoints, notably TNFRSF family members 4, 9, 14, 18, and CD27. Notably, the correlation with inhibitory CD276 exhibited the highest value among the checkpoints examined (cor = 0.387, *p* = .003) (Figure 4(e)). These findings indicate that KRT81 may affect immune cell infiltration in TNBC.

### KRT81 predicts the response of immunotherapy and chemotherapy sensitivities

**Figure 5.**
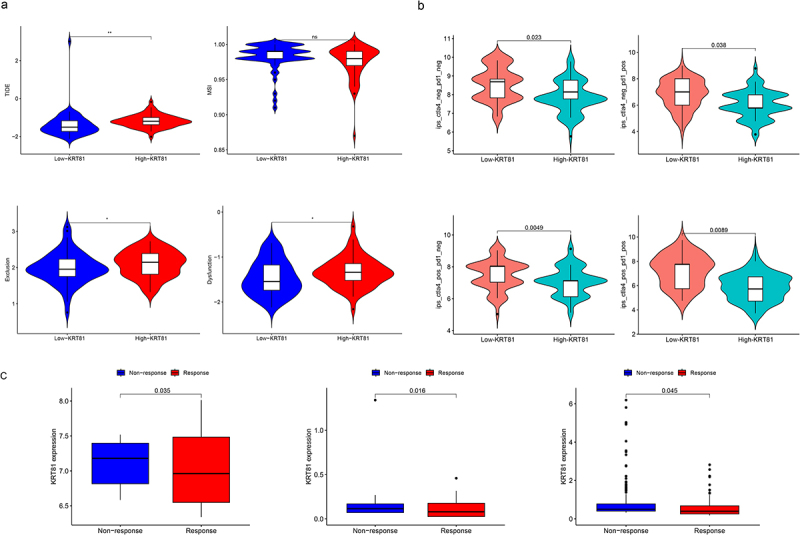
Validation of KRT81 as a predictor of immunotherapy response and drug sensitivity in TNBC patients. (a) The differences in TIDE, MSI, exclusion, and dysfunction scores between the high- and low-KRT81 groups. (b) The differences in IPS scores between the high- and low-KRT81 groups. (c) Comparing the expression levels of KRT81 in patients with non-response and response to immunotherapy. (d) Correlation analysis of KRT81 expression level and sensitivity of common chemotherapy drugs for TNBC.

Following that, we explored whether KRT81 was correlated to immunotherapy. TIDE score stands for the integrated score of tumor immune dysfunction and rejection, which is used to assess the possibility of tumor immune evasion in the gene expression profile of the tumor sample. The higher the TIDE prediction score, the higher the possibility of immune evasion, indicating that the patient is less likely to benefit from ICI treatment.^25^ We then calculated the TIDE score of each TNBC patient in the TCGA cohort, and compared the differences between the high- and low-KRT81 groups. We found that the high-KRT81 group had higher scores of TIDE (*p* = .018), exclusion (*p* = .042), and dysfunction (*p* = .029), which implied that the patient in the group may be more likely to escape from the ICI therapy (Figure 5(a)). Meanwhile, another evaluated score of immunophenotype score (IPS) is also a new biomarker of tumor immunotherapy response rate, a high IPS score represents high immunogenicity.^26^ The findings from our study demonstrated that patients with TNBC belonged to the high-KRT81 group exhibited decreased IPS scores (including ips_ctla4_neg_pd1_neg, ips_ctla4_neg_pd1_pos, ips_ctla4_pos_pd1_neg, and ips_ctla4_pos_pd1_pos) compared to their counterparts in the low-KRT81 group (Figure 5(b)). Furthermore, we found that in the three datasets of GSE67501 (*p* = .035), GSE78220 (*p* = .016), and IMvigor 210 (*p* = .045), the ICI non-response patients expressed higher KRT81 than those response (Figure 5(c)). Therefore, these outcomes implied that patients in the low-KRT81 group may respond better to immunotherapy. Chemotherapy is the most common treatment for TNBC. We explored the sensitivities of five regular chemotherapy drugs including cisplatin, docetaxel, doxorubicin, gefitinib, and paclitaxel via the pRRophetic package. As shown in Figure 5(d), the IC50 of cisplatin (*p* = .022) and doxorubicin (*p* = .013) were higher in the high-KRT81 group, but lower IC50 exhibited docetaxel (*p* = .028) and paclitaxel (*p* = .024). These results suggest that KRT81 is a potent marker for predicting the response of immunotherapy and chemotherapy in TNBC.

### KRT81 + malignant subtype correlates to poor prognosis in TNBC

**Figure 6.**
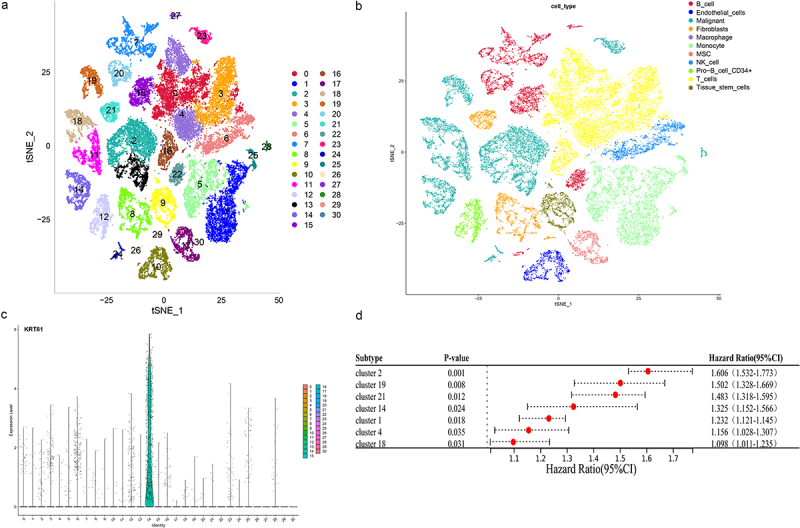
Analysis of the KRT81’s distribution in the cell subtypes by scRNA-seq GSE176078 dataset. (a, b) Visualization of cell clustering and annotation by t-SNE plots. (c) Expression of KRT81 in the different cell clusters. (d) Analysis of the relationship between the cell clusters and TNBC prognosis by univariate Cox regression.

Tumor is comprised of a complex mixture involving malignant tumor cells, immune cells, and stromal cells, showcasing both intra-tumor and inter-tumor heterogeneity [6]. The utilization of single-cell technology allows for a more thorough investigation into individual cells and molecules, leading to profound and detailed insights [7]. Nine samples of TNBC within the single-cell dataset GSE176078 were obtained to study the distribution of KRT81. Following the amalgamation of these samples using the Harmony and Seurat package, we generated PCA plots for each sample along with the visualization of the top 2000 highly variable genes (Figure S4a,b). Through this process, we successfully identified a sum of 31 clusters (Figure 6(a)), and the visualization of the top 5 marker genes can be observed in Figure S4c. A total of 11 cell types including B cells, endothelial cells, malignants, fibroblasts, macrophages, monocytes, mesenchymal stem cells (MSC), natural killer (NK) cells, CD34 + pro B cells, T cells, tissue stem cells were annotated (Figure 6(b)). We proceeded to visualize the expression pattern of KRT81 using tSNE and violin plots. This visualization distinctly revealed that KRT81 expression was concentrated within cluster 14, a subtype associated with malignancy (Figure 6(c)). Following that, our objective was to investigate potential enriched pathways specific to cluster 14, leveraging the genes that exhibited differences compared to the genes present in the other clusters. The GO annotation analysis unveiled associations with ATP synthesis coupled electron transport, mitochondrial ATP synthesis coupled electron transport, aerobic respiration, and oxidative phosphorylation (Figure S5a). Likewise, the outcomes from the KEGG analysis indicated the presence of pathways such as oxidative phosphorylation, carbon metabolism, glycolysis/gluconeogenesis, and the citrate cycle (TCA cycle) (Figure S5b). Moreover, we applied the univariate Cox to figure out seven clusters, including cluster 14 (HR = 1.325, 95% CI: 1.152–1.566, *p* = .0024) that were associated with poor prognosis in the TCGA dataset (Figure 6(d)). Collectively, these findings provide compelling evidence that KRT81 is specifically expressed within a subtype of malignant that predicted poor prognosis for TNBC patients.

### KRT81 promotes TNBC cell proliferation, migration, invasion, and adhesion

**Figure 7.**
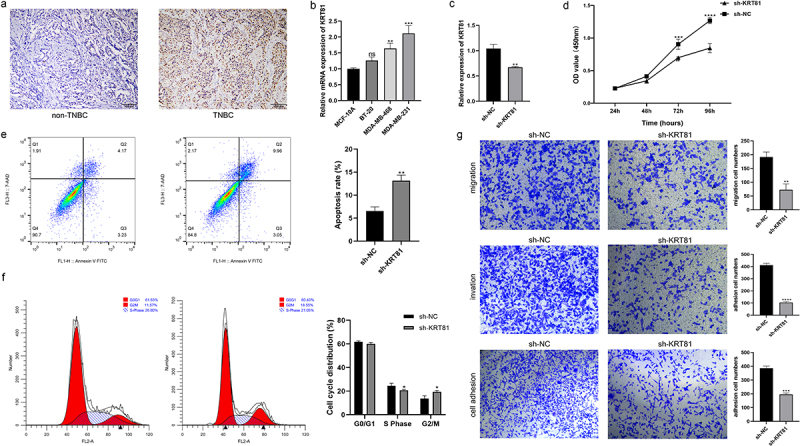
The effects of knocking down KRT81 on MDA-MB-231 cell line. (a) Verification of the KRT81’s expression in the clinical tissues by IHC, the non-TNBC tissue was obtained from the normal margin during the surgery. (b) The relative expression level of KRT81 of MDA-MB-231 treated with overexpressed or blank plasmid. (c) The proliferation curves of the MDA-MB-231 cell line. (d) Cell apoptosis rate between the sh-NC and sh-KRT81 groups. (e) The cell cycle distribution of MDA-MB-231. (f) Effects of knocking down KRT81 on cell migration (the upper), invasion (the middle), and adhesion (the lower), respectively.

**Figure 8.**
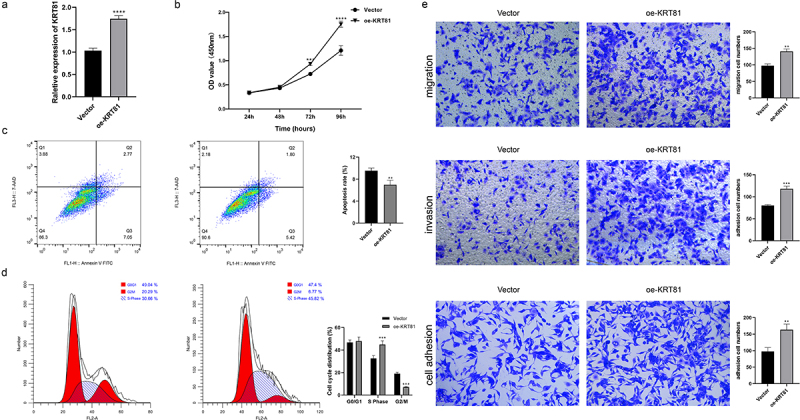
The effects of KRT81 overexpression on BT-20 cell line. (a) Relative expression level of KRT81 of BT-20 cell line treated with overexpressed plasmid or vector. (b) The proliferation curves of BT-20 cell line. (c) Cell apoptosis rate between the vector and oe-KRT81 groups. (d) The cell cycle distribution of BT-20 cell line. (e) Effects of overexpressed KRT81 on cell migration (the upper), invasion (the middle), and adhesion (the lower), respectively.

We subsequently assessed the expression of KRT81 in clinical tissues using IHC. The results revealed a significantly higher level of KRT81 expression in TNBC tissues compared to non-TNBC tissues (Figure 7(a)). To identify suitable TNBC cell lines for subsequent gene editing experiments aimed at validating the functional role of KRT81, we calculated the relative expression of KRT81 in three TNBC cell lines and one normal breast epithelial cell line. Among these, MDA-MB-231 exhibited the highest relative expression (*p* = .001), while BT-20 showed no statistical significance when compared to MCF-10A (Figure 7(b)). Consequently, MDA-MB-231 was chosen for gene knockdown, and BT-20 was selected for gene overexpression experiments. We successfully decreased the KRT81 expression in the MDA-MB-231 cell line by specific shRNA (*p* = .0018) (Figure 7(c)). It showed that the knockdown of KRT-81 resulted in reduced cell proliferation at appointed times (Figure 7(d)). Meanwhile, the cell apoptosis rate was higher in the sh-KRT81 group (13.14 ± 0.936) than in the sh-NC group (6.56 ± 0.862, *p* = .0016) (Figure 7(e)). The cell cycle was also affected. It showed that the knockdown of KRT81 led to cell arrest in the cycle of G2/M (Figure 7(f)). The GSEA pathway analysis showed that KRT81 is highly associated with focal adhesion, cell adhesion molecules cams, and ECM receptor interaction, we then tested the cell ability of migration, invasion, and adhesion. As shown in Figure 7(g), the ability of cell invasion, migration, and adhesion was weakened after the knockdown of the KRT81. On the other side, we genetically overexpressed KRT81 in the BT-20 cell line exhibited the opposite effect (*p* < .0001) (Figure 8(a)). Cell proliferation demonstrated a notable augmentation, concomitant with a reduction in cell apoptosis (Figure 8(b,c)). The distribution of cells in the G2/M cycle exhibited a marked decrease, whereas a substantial increase was observed in the proportion of cells in the S phase (Figure 8(d)). Further, the cell ability of migration, invasion, and adhesion was enhanced by overexpression of KRT81 (Figure 8(e)). It indicated that KRT81 may play a vital role in promoting TNBC progression.

### KRT81 contributes to the suppression of CD8 + T cells

**Figure 9.**
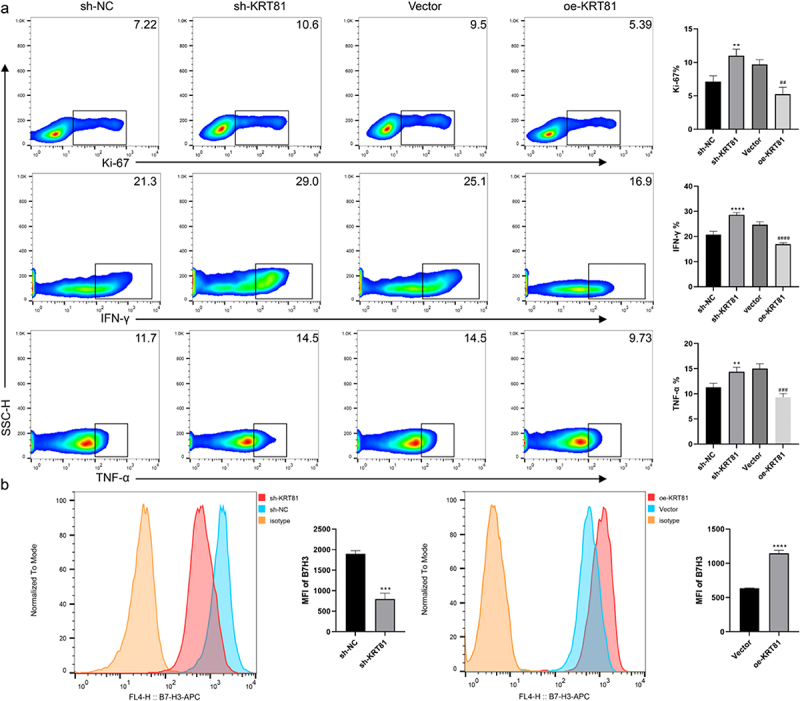
KRT81 is involved in suppressing CD8 + T cells via CD276 (a) Effects of gene alteration of KRT81 expression on proliferation (Ki-67) and cytokineS secretion (IFN-γ and TNF-α) of CD8 + T cells in co-culture system. (b) Effect of KRT81 knockdown on CD276 expression in MDA-MB-231 cell line (the left), Effects of overexpression of KRT81 on CD276 expression in BT-20 cell line (the right).

CD8 + T cells also referred to as cytotoxic T cells, play a crucial role in the immune system by recognizing and destroying cells that are infected or abnormal, including cancer cells.^27^ However, CD8 + T cells in the TNBC immune microenvironment are often affected by many different factors leading to anti-tumor dysfunction.^28^ Building on the results of the immune analysis, we conducted co-cultures of TNBC cells with PBMCs to investigate the potential involvement of KRT81 in immune suppression targeting CD8 + T cells. As shown in Figure 9(a), the downregulation of KRT81 in MDA-MB-231 cells alleviated the suppression of CD8 + T cells, resulting in enhanced proliferation of CD8 + T cells (ki67 positive ratio: 7.137 ± 0.858 vs 10.97 ± 1.002, *p* = .0038) and an increase in the expression of IFN-γ (20.7 ± 1.308 vs 28.6 ± 0.8718, *p* < .0001) and TNF-α (11.3 ± 0.781 vs 14.7 ± 0.9074, *p* = .0088). Conversely, overexpression of KRT81 in the BT-20 cell line led to a significant inhibition of CD8 + T cells, diminished cell proliferation, and a reduction in the expression of IFN-γ and TNF-α. Immunotherapy based on blocking immune checkpoints is a promising treatment for TNBC, CD276 has been raised as a promising marker for TNBC, and the expression of immune checkpoints is correlated with multiple factors.^24,29,30^ To verify whether the CD276 was affected by KRT81, we further detected the expression of CD276 on the cell surface. While knockdown of KRT81, the expression of CD276 was reduced (mean fluorescence intensity (MFI): 1901 ± 70.81 vs 798.7 ± 142.7, *p* = .0003), the effect was reversed in the oe-KRT81 group (MFI: 635.7 ± 4.163 vs 1149 ± 40.93, *p* < .0001) (Figure 9(b)). Thus, it suggested that KRT81 may suppress CD8 + T cells via regulating CD276 in TNBC.

## Methods and materials

### Data collection

Two datasets of TNBC were queried from The Cancer Genome Atlas (TCGA) (https://portal.gdc.cancer.gov/) and the Molecular Taxonomy of Breast Cancer International Consortium (METABRIC) (https://www.mercuriolab.umassmed.edu/metabric) databases. The keratin family genes were searched from the GenCard database (https://www.genecards.org/) with the keyword “keratin”, the gene with the category “protein-coding” and relevance score > 10.00 was retained as keratin-related genes, a total of 150 unique protein-coding genes were retained. For detection of the gene expression in the immunotherapy cohorts. Expression profiles stored in series matrix form in two immunotherapy datasets of GSE67501 and GSE78220 were downloaded from the Gene Expression Omnibus (GEO, https://www.ncbi.nlm.nih.gov/gds/?term=) via searching the GSE number. Further, the gene expression profile of the public immunotherapy cohort of IMvigor210 (http://research-pub.gene.com/IMvigor210CoreBiologies/) was also obtained.

### Screening of differentially expressed genes (DEGs)

We utilized the “limma” R package to discern DEGs among the high- and low-KRT81 in the TCGA. The differentially expressed genes (DEGs) that met the criteria of an adjusted *p*-value < .05 and an absolute log2 fold change (|log2 FC|) ≥ 1 were selected.

### Survival analysis

For analysis of the prognostic of KRT81 in the different subtypes of BC, we searched the survival curve by setting hormone receptor expression through the Kaplan-Meier plotter web page (https://kmplot.com/analysis/). Briefly, we searched the KM Plotter of RNA-seq for breast cancer, entered the gene symbol, and selected the corresponding status of hormone receptors ER, PGR, and HER2. The package “surviminer” was adopted to plot the survival curve based on the data from the TCGA and METABRIC.

### Cox regression analysis

Three regression analysis methods were used in this study. To obtain prognostic KRT-related genes, we applied the least absolute shrinkage and selection operator (LASSO) Cox regression to minimize overfitting via the “glmnet” package. To determine whether the KRT81 was an independent factor for predicting OS from the other clinical features, the function of univariate and multivariate Cox analysis in the “survival” package was employed.

### Functional annotation analysis

After identifying the DEGs between the high- and low-KRT81 groups, we utilized the “clusterProfiler” package to conduct gene ontology (GO) and Kyoto Encyclopedia of Genes and Genomes (KEGG) pathway enrichment analyses. To deepen our understanding of functional pathways with significantly enriched gene expression levels, we carried out gene set enrichment analysis (GSEA) by the MsigDB database (https://www.gsea-msigdb.org/gsea/msigdb/index.jsp).

### ESTIMATE scores and immune infiltration analysis

To investigate the differences in immune cell infiltration between the high-KRT81 and low-KRT81 groups, we utilized the ESTIMATE algorithm to compute the StromalScore, ImmuneScore, and ESTIMATEScore for each group. Then, the CIBERSORT algorithm for immune infiltration was applied with the “CIBERSORT” R package to assess the infiltration score of 28 immune cells and the proportion of annotated cell clusters in the TCGA cohort.

### Calculation of TIDE and IPS scores

To predict the response of immunotherapy, we compared the TIDE and IPS scores between high-KRT81 and low-KRT81 in different immunotherapy cohorts. We obtained the immunotherapy data from the tumor immune dysfunction and exclusion via uploading the expression profile of TNBC to the TIDE website (http://tide.dfci.harvard.edu), and the website would output the scores after calculating based on a specific algorithm. Based on the model gene expression we then evaluated MSI scores, dysfunction scores, exclusion scores, and the TIDE score. We directly downloaded the Immunophenoscores (IPS) of TNBC patients from the TCIA database (https://tcia.at/) by searching the program BRCA.

### Drug sensitivities analysis

CellMiner （https://discover.nci.nih.gov/cellminer/home.do）is a versatile query tool and database that serves as a valuable resource for studying and integrating pharmacological and molecular data derived from NCI-60 cancer cell lines. Transcriptome data from the website were retrieved for analysis. Spearman’s correlation analysis was executed to elucidate the association between drug sensitivity and gene expression, employing |cor| > 0.3 and a *p* < .01 as stringent criteria for statistical significance.

### Single-cell RNA sequencing GSE176078 analysis

The scRNA-seq dataset underwent processing using the “Seurat” package. Criteria for inclusion involved each gene being expressed in a minimum of 10 cells, with each cell expressing at least 200 genes. Cells with over 10% mitochondrial-expressed genes were excluded. Batch effects were corrected using the fastMNN algorithm, and the ‘FindVariableFeatures’ function identified the top 2000 highly variable genes. Dimensionality reduction was achieved through t-SNE analysis (resolution = 0.5). To screen for marker genes, the ‘FindAllMarkers’ function was employed, with |logFC >0.5| and an adjusted p-value < .05 serving as threshold criteria. Subsequently, we referenced the Human Primary Cell Atlas data and utilized the “SingleR” package for automatic annotation of the identified cell clusters. Visualization of these clusters was facilitated through t-SNE plots.

### Immunohistochemistry (IHC)

Breast cancer specimens, including normal areas which were the margin tissue, were obtained from patients at the time of surgery. The archival specimens were obtained from the Zhongshan Hospital of Traditional Chinese Medicine Affiliated to Guangzhou University of Chinese Medicine. The sections (4-µm) were mounted onto silane-coated slides. Immunohistochemistry was automatically performed using the Ventana XT System Discovery® (Roche). Briefly, tissue sections were treated with protease I (Roche) at 37̊C for 16 min for antigen retrieval. The following primary antibody was used: KRT81 polyclonal antibody was diluted at 1:25 (Abcam), and sections were incubated at 37̊C for 32 min. The tissue sections were then incubated at 37̊C for 20 min with a universal secondary antibody (Affinity). The site of peroxidase binding was determined using the DISCOVERY DABMap Detection kit (Roche). Sections were then counterstained with Hematoxylin II (Roche) for microscopic examination. As a negative control, nonimmune γ-globulin was used instead of the antibody. The specimens were observed and photographed using a fluorescence microscope FSX100 (Olympus).

### Cell culture and transfection

The human normal mammary epithelial cells MCF-10A and TNBC cell lines of BT-20, MDA-MB-468, and MDA-MB-231 (Procell) were grown at 37°C and 5% CO2 in RPMI1640 containing 10% fetal bovine serum (FBS, Gibco). The targeting KRT81 shRNA and negative control were synthesized by GenePharma. The sequences of sh-KRT81 and sh-NC were: 5′-ACCUGCGGAUCAGGAUUUGTT-3′ （sense）and 5′-CAAAUCCUGAUCCGCAGGUTT-3′ (anti-sense); 5′-UUCUCCGAACGUGUCACGUTT-3′ （sense）and 5′-ACGUGACACGUUCGGAGAATT-3′ (anti-sense), respectively. Lipofectamine 8000 (Beyotime) was used to transfect the siRNA constructs into the MDA-MB-231 cells. Cells were harvested for subsequent experiments 48 hours posttransfection. Full-length KRT81 DNA was cloned into the pcDNA3.1 (+) vector (GenePharma) and transfected to the BT-20 cell line by Lipofectamine 8000 for overexpression of KRT81.

### RNA extraction and quantitative real-time PCR (qPCR)

Total RNA was extracted from the cells using the RNeasy Mini kit (Qiagen). cDNA was reverse-transcribed from total RNA (200 ng) using the thePrimeScript™ RT Master Mix (Takara). PCR was performed with Takara LA Taq® with GC Buffer (Takara) using specific primer pairs. PCR amplification consisted of 30 sec at 94̊C, 30 sec at 55–60̊C, and 30 seconds min at 72̊C for 40 cycles. Gene-specific primers were designed according to known human sequences using the Primer3Plus software. The RT-PCR products were subjected to electrophoresis on a 2% agarose gel and visualized with ethidium bromide. Levels of specific mRNAs were quantified by qPCR using ChamQ SYBR qPCR Green Master Mix (Vazyme Biotech). Transcript levels were normalized to that of GAPDH cDNA. The primers used were as follows (5‘→3’): KRT81, F-AGGCTATGTGAAGGCATTGG (corresponding to exon 8) and R-AAGTGGGGGATCACACAGAG (corresponding to exon 9); GAPDH, F-AGAAGGCT GGGGCTCATTTG and R-AGGGGCCATCCACAGTCTTC. PCR specificity was ascertained by melting curve analysis. Relative gene expression was calculated according to the 2-ΔΔCt method.

### Cell counting kit-8 (CCK-8) assay

Cellular proliferation after transfection was measured by the CCK-8 assay (GLPBIO). Briefly, at a density of 1000 cells/well, cells were grown in 96-well plates containing DMEM with 10% FBS, following which 10 *μ*l CCK-8 solution was added per well at the indicated time points. These cells were incubated for 2 h, and the absorbance values at 450 nm were detected on a microplate spectrophotometer (ThermoFisher) to determine cellular proliferation.

### Cell apoptosis assay

Cell apoptosis was evaluated using the annexin Annexin-V-FITC/PI detection kit (KeyGen Biotech). The cells were collected and subjected to staining with annexin Annexin-V-FITC and PI for 10 minutes. Flow cytometry analysis was performed using FACSCalibur (BD Biosciences) to determine the rate of cell apoptosis. The obtained data regarding the apoptosis rate were quantified using FlowJo software (v10).

### Cell cycle analysis

For cell cycle analysis, transfected cells were harvested through centrifugation and subsequently fixed in 75% ethanol at 4°C for 24 hours. Following another round of centrifugation, the cells were stained with PI (KeyGen Biotech) for 20 minutes, and the resulting fluorescence was measured using flow cytometry. The acquired data were processed using Modfit LT software (v5.0) for further analysis of the cell cycle distribution.

### Cell migration and invasion assay

To evaluate the roles of KRT81 in the migration and invasion of TNBC cells, a 6.5 mm transwell (Corning) was employed. For the migration assay, 1 × 10^4^ cells were seeded in the upper chamber of the transwell. In the invasion assay, the transwell chamber was pre-coated with Matrigel dissolved in ABW BIO. The Matrigel was diluted with FBS-free medium at a ratio of 1:6, and then 1 × 10^5^ cells were added to the transwell chamber. After incubation, were fixed in 4% formaldehyde, stained with 0.1% crystal violet solution, and counted using a light microscope (Nikon).

### Cell adhesion assay

First coat the 96-well plate with 10 μg/ml fibronectin (Solarbio) at room temperature for 1 hour. Aspirate off the coating solution add 200 μl of heat-denatured 1% BSA and incubate the plate at 37°C for 1 hour. Wash the plate twice with a serum-free medium. After cell counting, 3 × 10^4^ cells were plated in a pretreated 96-well plate, cultured for 24 hours, and washed three times with PBS to remove non-adherent cells. After staining with crystal violet staining, the number of cells in the 96-well plate was detected under a microscope (Nikon).

### Isolation of peripheral blood mononuclear cells (PBMC) and co-cultured with tumor cell lines

The protocol of tumor cells co-culturing with PBMC was referred to in the article.^31^ Briefly, for isolation of PBMC from healthy volunteer samples, whole blood was mixed with the same volume of RPMI 1640, and then slowly spread on top of the Ficoll (Thermofisher). The mixture was centrifuged at 800 g (room temperature) for 30 min. After centrifugation, the plasma layer was sucked away, the PBMC layer (i.e., the white membrane layer) was carefully absorbed and transferred to a new tube, and the cells were washed with PBS three times. Tumor cells were initially seeded onto 24-well plates at a density of 1 × 10^5^ cells per well, and PBMC was introduced at a 1:5 ratio 24 hours later. The total culture system volume was maintained at 500 μl, with a stimulating agent comprising phytohemagglutinin (PHA, eBioscience) at a concentration of 5 μg/ml (stock solution: 1 mg/ml) applied for 48 hours. Each cell group received three replicate wells. Six hours before cell collection, a leukocyte activation cocktail (BioLegend) containing phorbol 12-myristate 13-acetate (PMA, 40.5 μM), ionomycin (669.3 μM), and Brefeldin A (2.5 mg/ml) was added. A 1 μl aliquot of the cocktail was introduced into the medium.

### Flow cytometry

Cells were collected and subjected to three washes with PBS to ensure cleanliness and integrity. For the cell surface marker CD8, flow cytometry antibodies (PerCP, eBioscience) were introduced and incubated away from light at room temperature for 30 mins. Subsequently, the cells underwent a thorough three-time wash. For intracellular marker staining, cells were fixed and permeabilized using the Foxp3/Transcription Factor Staining Buffer Set (eBioscience) and then stained with fluorochrome-conjugated antibodies, including anti-ki-67-PE, anti-IFN-γ-PE, and anti-TNF-α-FITC, all sourced from eBioscience, for 30 mins. Post-staining, 100 μl of PBS was added to each tube for cell resuspension and detected by BD FACSCalibur flow cytometer. The acquired data were quantified using FlowJo software (v10).

### Statistical analysis

Statistical analyses were performed using R software (v4.2.2) and GraphPad Prism (v8.0). Survival analyses employed the log-rank test on Kaplan-Meier survival curves to assess the impact of high- and low-expression risk models on patient overall survival (OS). Correlation analysis was conducted using Spearman’s method. The Wilcoxon test was employed to compare scores (TIDE, IPS) between groups. Student’s t-test was conducted to compare results of CCK-8, apoptosis, migration, invasion, cell adhesion, and the ratio of ki-67, IFN-γ, and TNF-α. Data were presented as means ± standard deviation (SD). A significance level of *p* < .05 was considered statistically significant.

## Discussion

Among the diverse subtypes of breast cancer, TNBC distinguishes itself with a heightened metastasis rate, unfavorable prognosis, and diminished patient survival, solidifying its status as the most perilous subtype.^19^ The absence of effective treatments targeting TNBC accentuates the urgency in uncovering its molecular intricacies and identifying potential therapeutic targets. The anticipation for insights into the molecular mechanisms governing TNBC remains high, promising crucial advancements in the development of targeted therapies. In the present study, we collected bulk- and single-cell public datasets and applied comprehensive bioinformatic algorithms to identify KRT family members 81 that not only correlate to prognosis and immune infiltration but also could predict the response of immunotherapy for TNBC patients. Moreover, comprehensive experimental assessments were carried out to authenticate the pivotal role of KRT81 in governing the diverse biological behaviors of proliferation, migration, invasion, adhesion, and its intricate association with CD8 + T cells within TNBC cells.

The KRT family encompasses a set of genes responsible for encoding keratins, a class of fibrous structural proteins that are the key components of intermediate filaments in epithelial cells, providing strength and resilience to these cells.^6^ Different members of the KRT family may be expressed in different tissues or during specific stages of development. Mutations or dysregulation of KRT genes can be associated with various genetic disorders affecting the skin and other epithelial structures.^6,32^ We defined 11 KRT family members correlated to the prognosis of TNBC via Cox regression analysis, while the KRT81 exhibited the highest risk value among these genes. KRT81 has been reported to be increased in breast cancer.^33,34^ However, we observed that the prognostic significance of KRT81 varies among breast cancer subtypes, its expression level was upregulated in TNBC tissues but not in non-TNBC tissues. Importantly, in comparison to other types of breast cancer, the hazard ratio of KRT81 for TNBC was the highest, suggesting that KRT81 could be a valuable prognostic factor for TNBC. The results of ROC curves and univariate Cox regression independent analysis further validated that KRT81 exhibited excellent ability for predicting the OS of TNBC patients.

TNBC is marked by a high invasion and metastasis rate and frequently experiences early-stage distant metastasis.^3^ Our investigation revealed a correlation between elevated KRT81 expression and distant metastasis. We conducted gene annotation to clarify the involved molecular pathways of KRT81 in TNBC. It showed that KRT81 was highly correlated with tumor metastasis-related signaling pathways such as focal adhesion, ECM receptor interactions, and cell adhesion molecules, suggesting it may promote tumor metastasis. Furthermore, *in vitro* experiments confirmed that the knockdown of KRT81 diminished the ability of TNBC cell migration, invasion, and adhesion. These results implied that targeted KRT81 may effectively inhibit invasion and metastasis of TNBC. Results from functional enrichment analysis also suggested a negative association between KRT81 and drug metabolism pathways, indicating a potential correlation to chemotherapy. We calculated the IC50 of five chemotherapy drugs (cisplatin, docetaxel, doxorubicin, gefitinib, and paclitaxel) between the high- and low-KRT81 groups. It indicated that the patients in the high-KRT81 group may be resistant to cisplatin and doxorubicin, but sensitive to docetaxel and paclitaxel. Detecting the expression level of KRT81 may guide the use of chemotherapy drugs for TNBC patients.

The immune microenvironment constitutes a vital component of cancer, exerting a pivotal role in the initiation and advancement of tumors.^16^ Understanding the cancer immune microenvironment is crucial for comprehending how tumors evade immune surveillance and for developing effective immunotherapies. Factors like the presence of immune cells, their activation status, and the immunosuppressive mechanisms within the tumor microenvironment play a pivotal role in shaping the immune response against cancer cells.^17^ Analyzing the cancer immune microenvironment could help researchers and clinicians identify potential therapeutic targets and improve the outcomes of immunotherapeutic interventions for cancer treatment. In this study, we identified KRT81 as a potential regulator influencing the TNBC immune microenvironment. It exhibited a positive correlation with tumor-infiltrating lymphocytes (TIL) of Tfh cells but displayed a negative correlation with CD8 + cytotoxic T cells. Notably, tumor cell lines with KRT81 knockdown demonstrated a diminished capacity to inhibit CD8 + T cells, suggesting that elevated levels of KRT81 promote an immunosuppressive microenvironment in TNBC. This effect may be associated with the immune checkpoint CD276, which showed a positive correlation with KRT81 expression. CD276 also referred as B7-H3, Yang et al. reported that abnormal N-glycosylation of B7H3 participated in suppressing the immunological function of CD4 + and CD8 + T cells.^35^ The underlying mechanism of how KRT81 regulates CD276 expression remains to be explored. Inflammatory cytokine IL-8 plays a major role in recruiting polymorphonuclear myeloid-derived suppressor cells (PMN-MDSCs), which inhibit the anti-cancer function of T cells in TNBC.^36^ Zhang et al. found that the expression of IL-8 was reduced after the knockdown of KRT81 in melanoma cells inhibited the progression of the tumor.^37^ This may also be one of the ways that KRT81 participates in tumor immunosuppression, and further studies are needed.

Chemotherapy serves as the primary treatment for TNBC, but its efficacy is constrained. In recent years, immunotherapy targeting immune checkpoints (PD-1/PD-L1, CTLA-4) has emerged as a promising treatment modality for TNBC.^38^ However, not all TNBC patients respond efficiently to the treatment. It is imperative to identify predictive markers to categorize responsive patients and enhance the effectiveness of immunotherapy. The TIDE scoring system, which stands for tumor immune dysfunction and rejection, is employed to assess the prospective clinical efficacy of immunotherapy in distinct risk groups, providing insights into the tumor’s potential to evade immune responses.^25^ To predict the response to immunotherapy in TNBC, we compared TIDE scores between high- and low-KRT81 groups, finding higher scores in patients with elevated KRT81 levels, indicating a potential inadequate response to immunotherapy. The IPS scoring system was also employed, revealing lower scores in the high-KRT81 group, suggesting the potential ineffectiveness of PD-1/PD-L1 or CTLA-4 blockade therapy. Furthermore, non-responsive patients exhibited significantly higher KRT81 expression levels than responsive patients. These findings implied that KRT81 could be an effective predictor of immunotherapy response in TNBC, possibly linked to its involvement in the immunosuppression of CD8 + T cells. Genetic deletion or reduction of KRT81 at the tumor site may enhance the efficacy of immunotherapy for TNBC.

Bulk RNA-sequencing assesses the average gene expression level across the entire tissue sample, whereas scRNA-sequencing examines gene expression at the single-cell level that effectively addresses the challenge of cellular heterogeneity, a limitation in conventional transcriptome sequencing. Its application is expected to significantly advance medical and biological research.^39^ In this study, we analyzed the TNBC scRNA-seq sequencing dataset GSE176078 to detect the distribution and location of KRT81. A total of 31 subtypes were annotated as 11 cell subpopulations via the “Human Primary Cell Atlas data” in the “SingleR” package. Gul and colleagues identified 21 distinct cell clusters, with a clustering resolution value of 0.4.^40^ To explore additional tumor subpopulations, the resolution was increased to 0.5 in our study. Therefore, the results yielded inconsistencies. How to choose the appropriate resolution value is an issue that still needs to be discussed and needs to be set according to the research purpose. The specific localization of KRT81 within a malignant subtype was observed, and univariate Cox regression analysis indicated a correlation between the KRT81 + malignant cluster and worse survival in TNBC. Furthermore, the KRT81 + malignant cluster emerged as a predictor for various cancers, including sarcoma (SARC), acute myeloid leukemia (LAML), mesothelioma (MESO), ovarian cancer (OV), cervical cancer (CESC), prostate cancer (PRAD), and lung adenocarcinoma (LUAD).^41,42^ These findings suggested that KRT81 may serve as a pan-cancer prognostic marker.

The present study still has some limitations. Data in the study was sourced from TCGA and METABRIC datasets. While these datasets provide valuable information, they may have limitations such as biases, variations in sample collection, or incomplete clinical information that could impact the robustness and generalizability of the findings. The clinical correlation was based on retrospective analyses, and the predictive value of KRT81 for immunotherapy and chemotherapy response should be validated in prospective clinical studies with larger patient cohorts. While the study suggests a potential role of KRT81 in immune suppression and CD8 + T cell function, the underlying molecular mechanisms and signaling pathways involved are not extensively explored. The correlation between KRT81 and immune suppression, especially CD8 + T cells function, was assessed *in vitro*. *In vivo* studies by building a mouse model or analysis of patient samples could provide more clinically relevant insights into the interaction between KRT81 and the immune microenvironment. Further mechanistic studies are needed to elucidate the detailed pathways through which KRT81 influences TNBC progression and immune responses.

## Supplementary Material

Figure S1.tif

Figure S3.tif

Graphical abstract.tiff

Figure S5.tif

Figure S4.tif

## Data Availability

The sequencing data could be sourced from the mentioned public database. The corresponding authors can be contacted with questions about additional information in this study
