# Supplementary figures and images for: The prognostic marker KRT81 is involved in suppressing CD8 + T cells and predicts immunotherapy response for triple-negative breast cancer
Source: Cancer Biol Ther. 2024 May 22;25(1):2355705. doi: 10.1080/15384047.2024.2355705 (PMC11123506; doi:10.1080/15384047.2024.2355705)

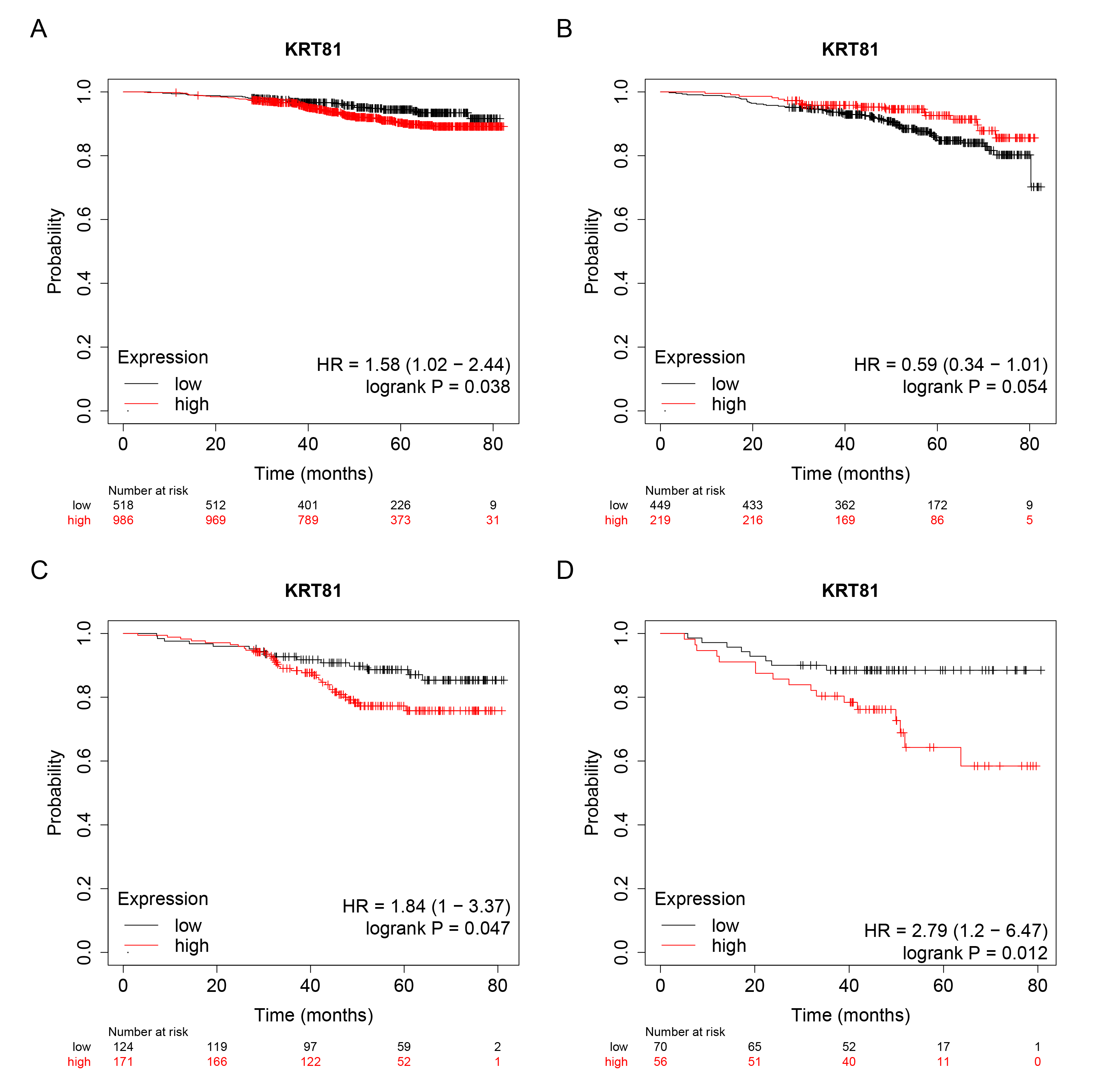

Supplement: Figure S1.tif [file KCBT_A_2355705_SM6695.tif]

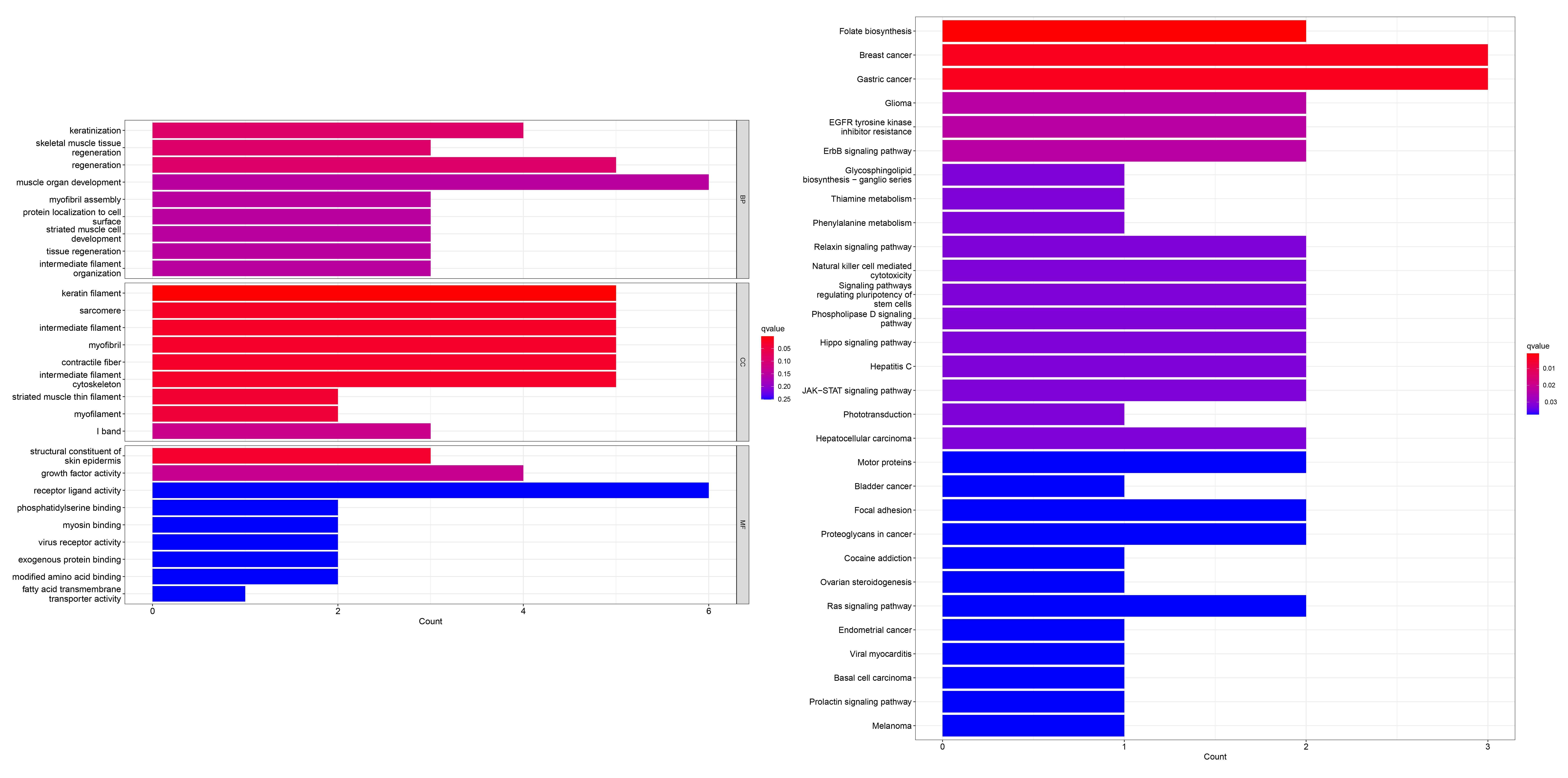

Supplement: Figure S3.tif [file KCBT_A_2355705_SM6694.tif]

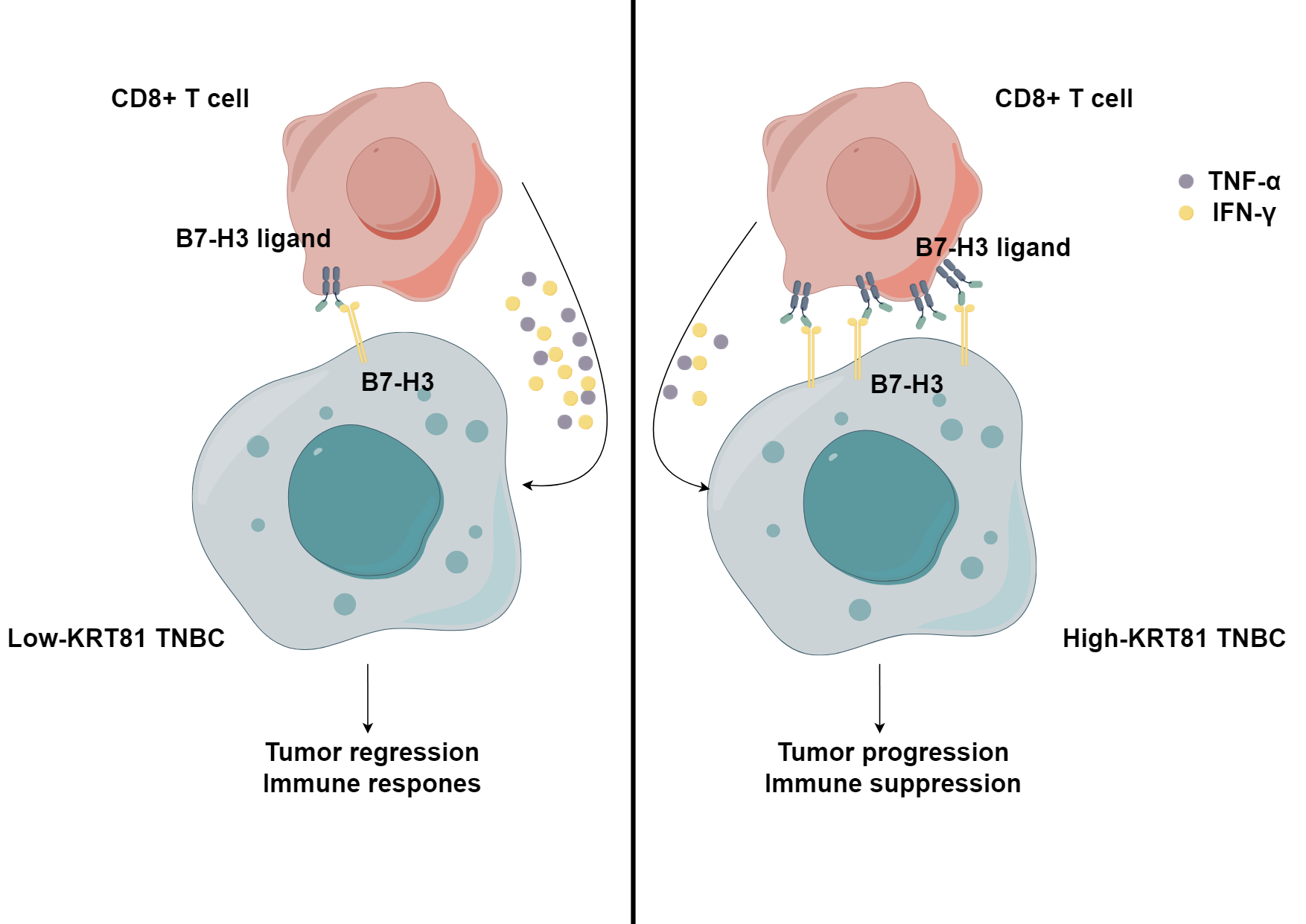

Supplement: Graphical abstract.tiff [file KCBT_A_2355705_SM6693.tiff]

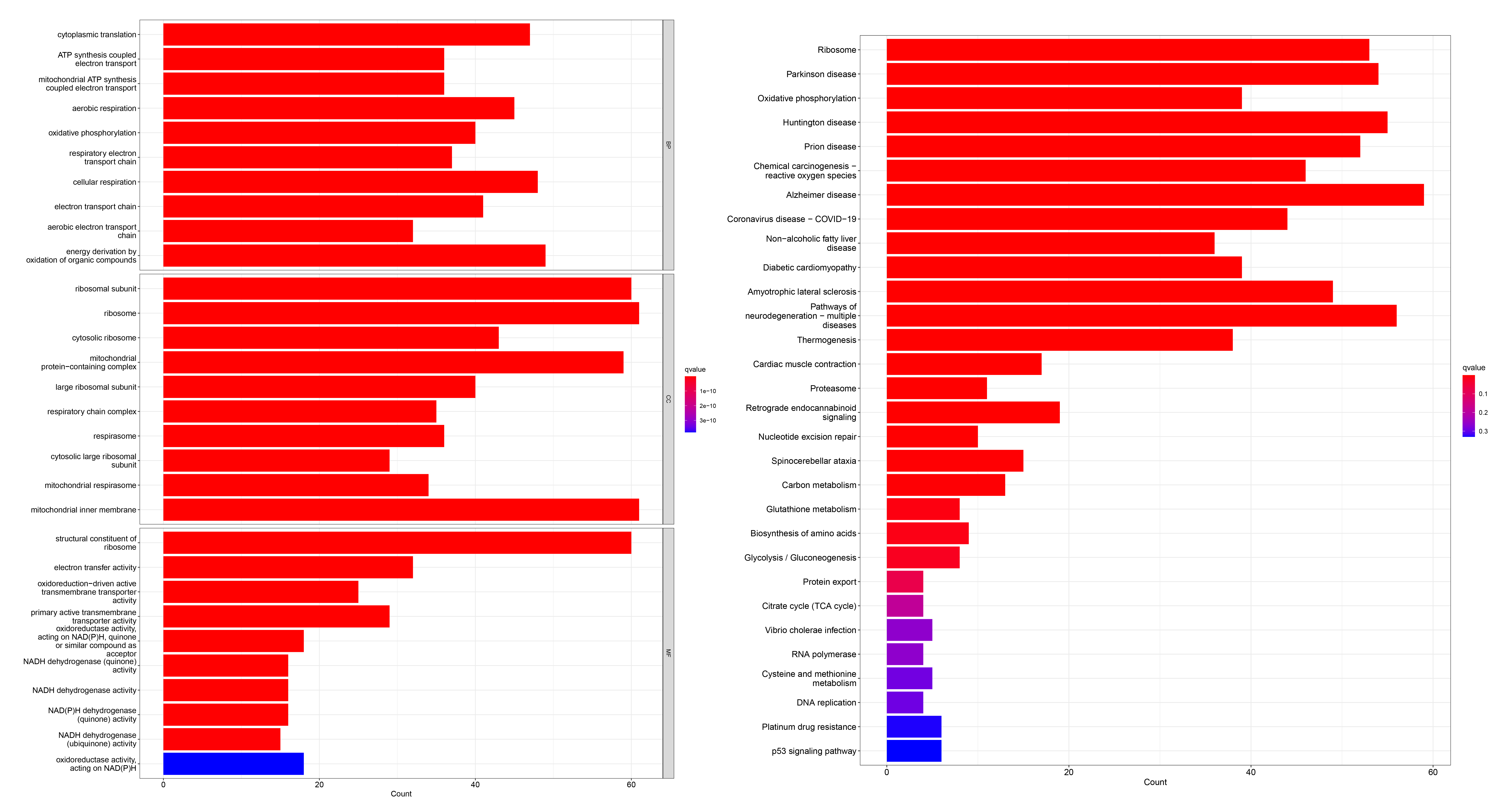

Supplement: Figure S5.tif [file KCBT_A_2355705_SM6692.tif]

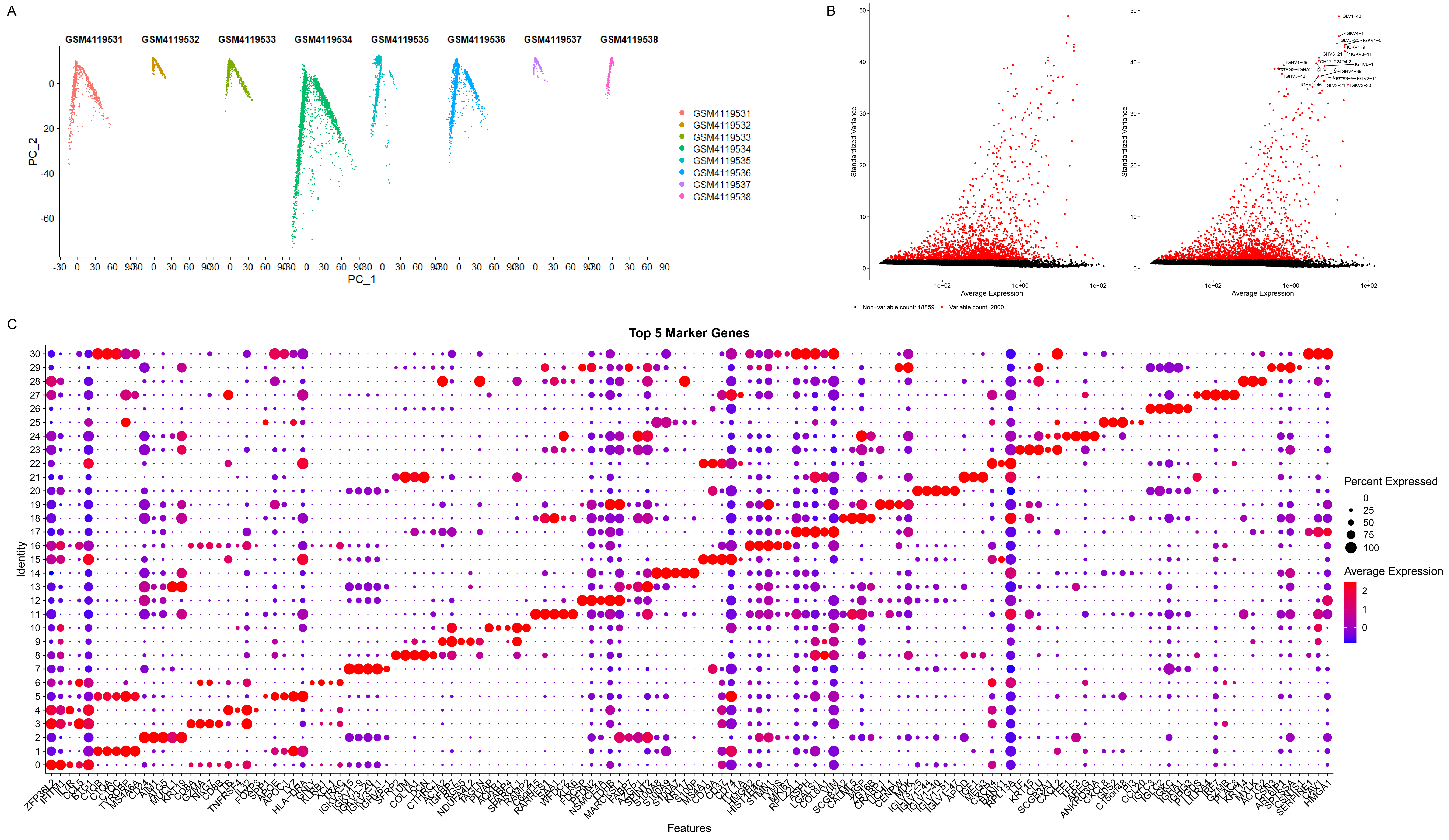

Supplement: Figure S4.tif [file KCBT_A_2355705_SM6691.tif]
